# On-Board Event-Based State Estimation for Trajectory Approaching and Tracking of a Vehicle

**DOI:** 10.3390/s150614569

**Published:** 2015-06-19

**Authors:** Miguel Martínez-Rey, Felipe Espinosa, Alfredo Gardel, Carlos Santos

**Affiliations:** Department of Electronics, University of Alcalá. Polytechnic School, Campus Universitario, Alcalá de Henares 28871, Spain; E-Mails: felipe.espinosa@uah.es (F.E.); alfredo@depeca.uah.es (A.G.); carlos.santos@depeca.uah.es (C.S.)

**Keywords:** event-based state estimation, indoor localization, non-linear filtering, trajectory tracking

## Abstract

For the problem of pose estimation of an autonomous vehicle using networked external sensors, the processing capacity and battery consumption of these sensors, as well as the communication channel load should be optimized. Here, we report an event-based state estimator (EBSE) consisting of an unscented Kalman filter that uses a triggering mechanism based on the estimation error covariance matrix to request measurements from the external sensors. This EBSE generates the events of the estimator module on-board the vehicle and, thus, allows the sensors to remain in stand-by mode until an event is generated. The proposed algorithm requests a measurement every time the estimation distance root mean squared error (DRMS) value, obtained from the estimator's covariance matrix, exceeds a threshold value. This triggering threshold can be adapted to the vehicle's working conditions rendering the estimator even more efficient. An example of the use of the proposed EBSE is given, where the autonomous vehicle must approach and follow a reference trajectory. By making the threshold a function of the distance to the reference location, the estimator can halve the use of the sensors with a negligible deterioration in the performance of the approaching maneuver.

## 1. Introduction

Recent years have witnessed a growing interest in the use of event-based communications for cyber-physical systems. One example is autonomous vehicle guidance [1] in intelligent spaces [2]. In these environments, sensors often have to cope with scarce resources, such as communication bandwidth and processing capacity. In addition, the sensors are often battery-powered, so it is necessary to optimize all sensor functions in order to extend battery life.

Several strategies have recently been developed to reduce the number of measurements used by the estimator and transmitted through the network channel. Most of them rely on the send-on-delta (SoD) method, also known as Lebesgue sampling [3,4]. According to this method, a sample is sent to the estimator whenever the measured value exceeds certain limits with respect to the previous sample sent.

Many variants of the same principle have been proposed. In [5], the authors study and compare some SoD extensions that include integrating the difference between the current sensor value and the last sample transmitted (send-on-area) or integrating this difference squared (send-on-energy). In [6], a predictor for the expected next sample is used based on the previous samples. In addition, in [7], a delta variable is set in the presence of disturbances.

In all of the above-mentioned triggering schemes, the sensors get to decide when a sample should be sent to the estimator. This implies that the sensor must be continuously running and monitoring the measured variable in order to detect the event. Meanwhile, the estimator at the other side of the communication channel waits for the measurements and uses them when they arrive. Thus, this kind of estimator is called an event-based state estimator (EBSE).

These are distributed estimator systems, because the event is generated by the sensor module rather than by the estimator. Along the same lines, some authors have explored the implementation of distributed Kalman filters, whereby each sensor node runs its own filter with the information that it is capable of sensing. The nodes communicate with each other to achieve a common estimation and error covariance matrix [8–11].

Some authors have proposed estimators that can refine their estimation even in the absence of information from the sensors. Each triggering criterion defines a region where the sensed signal must lie when there are no updates from the sensors. In [12], a Kalman filter is applied assuming a uniform probability for the aforementioned region. However, the Kalman filter assumes a Gaussian density function for the measurement noise, and hence, a sum of Gaussians to approximate the probability of the region is proposed in [13]. The authors of [14] provide a method to obtain the optimal gain for an estimator with point- and set-valued measurements. This concept is extended in [15], where the minimum mean-squared error (MMSE) estimator is obtained for multiple sensors. The maximum likelihood estimator is also developed in [16].

If event generation is performed independently from the sensed signal, the sensors can be maintained in a standby state. Scheduling of the sample times would thus be performed by a centralized estimator that requests measurements from the sensors when they are necessary.

In controlled systems, there exist several works that perform the sampling in relation to the stability of the system based on a Lyapunov function [17–19]. However, the authors of these papers assume perfect measurements, and hence, estimator uncertainties are neglected.

The performance of an estimator is often evaluated by its estimation error covariance, so it is logical to consider using it to generate sensing events. In [20], the covariance matrix is used to determine an optimal schedule in a heterogeneous sensor network. In [21], a sensor uses the Kullback–Leibler divergence of the estimation error to decide whether a measurement should be sent. In the field of robot localization, in [22], the magnitude of the estimation error covariance is used by the estimator in to choose between using inertial measurements or the GPS signal.

Use of a triggering criterion based on the estimation error covariance for requesting a measurement is also analyzed in [23,24]. In these papers, the authors apply this scheme to linear systems, and for stationary problems, they typically observe convergence to periodic sampling. This convergence is proven for the special case of an unstable scalar system under some conditions.

The contribution of the present paper resides in the combination of a variance-based EBSE with an unscented Kalman filter (UKF) applied to the localization of an autonomous vehicle using external sensors, yielding a system that has the capacity to adapt to the maneuvers performed by the vehicle. As this is a non-linear system, the use of variance-triggered measurements does not make the EBSE converge to periodic solutions (unlike in [24]), and hence, sampling times must be computed online by the estimator module. Since the estimator module located in the vehicle decides the sampling times, the sensors can be maintained in standby, saving energy, bandwidth and processing power.

In a preliminary work [25], the triggering condition was obtained by evaluating the error variance of each state independently. The error variance of each state was maintained below a bounding value by requesting measurements every time the uncertainty of a state approached the bounding value. Here, we propose a new triggering condition that can be used with vehicle guidance control algorithms. The two state variables that represent the position in the Cartesian coordinate system are combined to obtain a single triggering condition, which is related to the estimation distance error.

The advantage of working with a distance error is that it provides a more meaningful and easier to tune threshold value that can be set according to the circumstances. In this case, the proposed EBSE is used in combination with a guidance control algorithm. The triggering threshold tightens as the vehicle approaches the trajectory to be tracked. As a result, many measurements can be prevented, because they are not necessary to fulfil the guidance task.

The remaining part of the paper is structured as follows: Section 2 presents a description of the system under study and the mathematical background used by the estimator and explains how the estimation error covariance is propagated. The contribution of the paper is located in Section 3, which introduces the concept of covariance-triggered measurements and the proposed adaptive distance error threshold. In Section 4, the proposed EBSE is tested by running a simulation, and the results obtained are discussed. Finally, some conclusions are drawn in Section 5.

## 2. Problem Description

**Figure 1:**
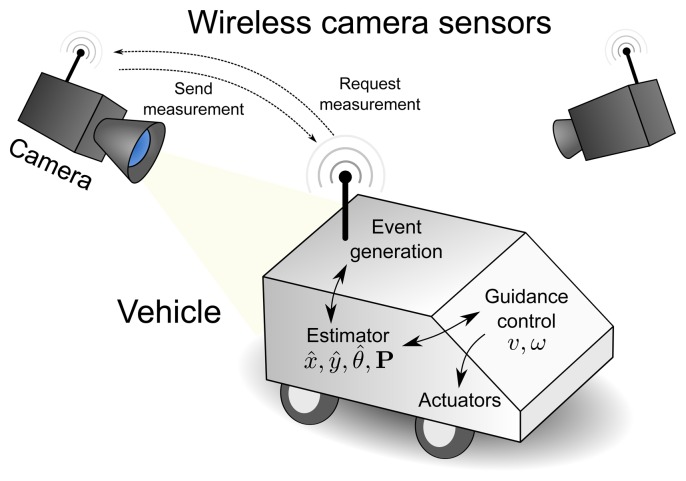
General description of the system showing the most important elements: the vehicle (controller and estimator) and sensors linked by a wireless network.

This paper deals with the localization and guidance of an autonomous vehicle using a state-space model and an external sensorial system based on cameras. Figure 1 depicts the main elements of the system. In the center of the figure, there is the autonomous vehicle that executes an estimation algorithm, as well as a guidance control to follow a pre-configured reference path. Above it, camera sensors that detect the position are connected to the vehicle via a wireless network. The technology used for the external sensors is not relevant, since the proposed method would work with any other kind of localization sensors, such as lasers, ultrasound or infra-red local positioning systems.

The sensors are only active when a measurement is requested. When a request is received, the corresponding camera activates and takes its measurement at the desired time. Then, the camera sends the measurement back to the vehicle, where it is processed to refine its pose estimation.

On-boarded sensors, such as wheel encoders or inertial measurement units, could be also used to refine the estimation, as in [25]. However, this paper does not consider any of these sensors in order to focus on the event generation of the remote sensors.

### 2.1. Mathematical Background

The system states are the coordinates *x* and *y* of the vehicle and its orientation angle θ. The continuous-time kinematic equations of the vehicle are as follows:

(1) $$\begin{array}{l} \dot{x}=\upsilon \cos\theta \\ \dot{y}=\upsilon \sin\theta \\ \dot{\theta }=\omega \end{array}$$

The symbols *υ* and ω are the system inputs and represent linear and angular velocity, respectively. The input vector **u**_c_ = [*υ*_c_ ω**_c_**]^T^ is the combination of the speed commands provided by a guidance algorithm. We consider that there might be uncertainties on the actual speeds due to model inaccuracies and input noises. These uncertainties are modeled as a zero-mean Gaussian random processes added to the input commands. The actual speeds of the system are then expressed as:

(2) $$\begin{array}{c} \upsilon =\upsilon_{\text{c}}+\Delta \upsilon \\ \omega =\omega_{\text{c}}+\Delta \omega \end{array}$$

The above-mentioned random noise has a covariance matrix **Σ_u_**:

(3) $$E\left[{\left[\begin{array}{cc} \Delta \upsilon & \Delta \omega \end{array}\right]}^{\text{T}}\left[\begin{array}{cc} \Delta \upsilon & \Delta \omega \end{array}\right]\right]=\sum_{\text{u}}$$

The system described in Equation (1) can be summarized in vector form:

(4) $$\dot{\text{x}}=\text{f}\left(x,\text{u}\right)$$

where *x* = [*x y* θ]^T^ ∈ *χ* ⊂ ℝ^3^ is the state vector, **u** = [*υ* ω]*^T^* ∈ 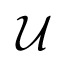 ⊂ ℝ^3^ is the input vector and *χ* and 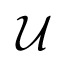 are the sets of all possible state points and inputs.

In practice, the system is controlled by a digital system that executes its algorithms periodically with a sample time *T*. To do so, the continuous-time system Equation (1) must be discretized, for which we propose the second order Runge-Kutta method [26].

(5) $$\begin{array}{l} x\left(t+T\right)=x\left(t\right)+T\upsilon \left(t\right)\cos\left(\theta \left(t\right)+\frac{T}{2}\omega \left(t\right)\right)\\ y\left(t+T\right)=y\left(t\right)+T\upsilon \left(t\right)\sin\left(\theta \left(t\right)+\frac{T}{2}\omega \left(t\right)\right)\\ \theta \left(t+T\right)=\theta \left(t\right)+T\omega \left(t\right) \end{array}$$

The discrete system above approximates the continuous-time system Equation (1) by turning the derivatives into difference equations. The system inputs are computed by the control module at discrete times *T*, 2*T*, 3*T*, *etc.*, and remain constant within the period *T*.

Equation (4) becomes:

(6) $$\text{x}\left(t+T\right)=f_{d}\left(\text{x}\left(t\right),u\left(t\right)\right)$$

The measured states are the *x* and *y* coordinates plus some measuring noise. They are obtained by a sensorial system at asynchronous discrete instants *t_k_*. Although these instants are assumed to be a multiple of the sample time *T*, they are not necessarily periodic and are scheduled by the estimator's event generator. The symbol *t_k_* represents the time of the *k*-th measurement:

(7) $$\begin{array}{ccc} t_{k}=n_{k}T & n_{k}\in \mathbb{N}, & n_{k}<n_{k+1}\forall k \end{array}$$

The output equation of the system is assumed to be a linear equation:

(8) $${\text{y}}_{\text{k}}={\text{Hx}}_{\text{k}}+{\text{w}}_{k}$$

where:

(9) $$\text{H}=\left[\begin{array}{lll} 1 & 0 & 0\\ 0 & 1 & 0 \end{array}\right]$$

The *k* suffix applied to a variable denotes the index of an asynchronous event. x*_k_* and y*_k_* are short forms for x(*t_k_*) and y(*t_k_*). w*_k_* is an uncorrelated random discrete noise vector with covariance matrix:

(10) $$E\left[{\text{w}}_{\text{k}}{\text{w}}_{\text{k}}^{\text{T}}\right]={\text{R}}_{\text{k}}$$

A Kalman filter can compute the estimated state vector x̂, as well as the estimation error covariance matrix **P**.

(11) $$\hat{\text{x}}=\text{E}\left[x\right]$$

(12) $$\text{P}=\text{E}\left[\left(\text{x}-\hat{\text{x}}\right){\left(x-\hat{x}\right)}^{\text{T}}\right]=\left[\begin{array}{ccc} \sigma_{x}^{2} & \sigma_{x,y} & \sigma_{x,\theta }\\ \sigma_{y,x} & \sigma_{y}^{2} & \sigma_{y,\theta }\\ \sigma_{\theta ,x} & \sigma_{\theta ,y} & \sigma_{\theta }^{2} \end{array}\right]$$

Let *e_x_* and *e_y_* be the estimation errors for both *x* and *y* coordinates. These are correlated random variables with zero mean. Let *P_i,j_* be the *i*-th row and *j*-th column entry of **P**. Then,

(13) $$\begin{array}{c} P_{1,1}=E\left[{\left(x-\hat{x}\right)}^{2}\right]=E\left[e_{x}^{2}\right]\\ P_{2,2}=E\left[{\left(y-\hat{y}\right)}^{2}\right]=E\left[e_{y}^{2}\right]\\ P_{1,2}=P_{2,1}=E\left[\left(x-\hat{x}\right)\left(y-\hat{y}\right)\right]=E\left[e_{x}e_{y}\right] \end{array}$$

Unfortunately, the Kalman filter is only optimal for linear systems affected by white Gaussian noise processes. The Gaussian probability density functions of the noise propagated through non-linear systems render the density function of the estimated state non-Gaussian, and iterating this non-Gaussian distribution over time becomes an intractable problem.

For a non-linear system, there are some algorithms that extend the idea of the Kalman filter, such as the extended Kalman filter (EKF) and the unscented Kalman filter (UKF) [27]. In both cases, the computed mean of the estimation x̂ and the estimation error covariance matrix **P** are not exact, but approximations.

Since Equation (1) is a non-linear set of equations, in our case, the estimation of the state vector is performed with the UKF. Although its computational cost is heavier, it provides a better approximation of P than the EKF [27].

There are two different stages for Kalman filter estimators. The prediction stage takes into account the plant model equations and the known inputs to advance the estimated point over time. The correction stage updates the estimation with the information obtained from a sensor measurement.

### 2.2. Prediction Stage

In this stage, x̂ must be propagated through the set of non-linear Equations (5). This is achieved by the unscented transformation [28]. The prediction stage is executed periodically for every *T* step. Because this stage does not require any information from the real world, It can be executed in real time or not. After receiving a measurement, the prediction for an arbitrary time span can be calculated in advance.

For each time step, a set of sigma-points are generated around the current estimated state point x̂ and are spread out according to **P**. The sigma points are state points that sample the probability density function of the state. In order to also take into account the input uncertainties, the state vector and the error covariance matrix are augmented with the mean and covariance of the noise.

(14) $$\begin{array}{cc} {\hat{x}}_{a}={\left[\begin{array}{ccc} {\hat{x}}^{\text{T}} & 0 & 0 \end{array}\right]}^{\text{T}}, & {\text{P}}_{a}=\left[\begin{array}{cc} \text{P} & 0\\ 0 & \sum_{\text{u}} \end{array}\right] \end{array}$$

A total of 2*N* sigma-points ${\hat{x}}_{a}^{\left(i\right)}$ are calculated with the formulas:

(15) $$\begin{array}{c} \begin{array}{cc} {\hat{x}}_{\text{a}}^{\left(i\right)}\left(t\right)={\hat{x}}_{\text{a}}\left(t\right)-{\left(\sqrt{NP_{a}\left(t\right)}\right)}_{i}^{T} & i=1,2,\ldots ,N \end{array}\\ \begin{array}{cc} {\hat{x}}_{a}^{\left(N+i\right)}\left(t\right)={\hat{x}}_{a}\left(t\right)-{\left(\sqrt{NP_{a}\left(t\right)}\right)}_{i}^{T} & i=1,2,\ldots ,N \end{array} \end{array}$$

where *N* is the number of states of the augmented state vector; so, in this case, *N* = 5 (three states plus two input noises), and ${\left(\sqrt{NP_{a}}\right)}_{i}$ is the *i*-th row of the Cholesky decomposition of matrix *N***P***_a_*.

The next estimated point is obtained as the mean of the sigma-points transformed by the discrete system function *f*_d_:

(16) $${\hat{x}}^{\left(i\right)}\left(t+T\right)=f_{\text{d}}\;\left({\hat{x}}_{a,1:3}^{\left(i\right)}\left(t\right),u_{c}\left(t\right)+{\hat{x}}_{a,4:5}^{\left(i\right)}\left(t\right)\right)$$

(17) $$\hat{x}\left(t+T\right)=\frac{1}{2N}\sum_{i=1}^{2N}{\hat{x}}_{a}^{\left(i\right)}\left(t+T\right)$$

and the covariance matrix as the cross-covariance of the transformed sigma-points:

(18) $$\text{P}=\frac{1}{2N}\sum_{i=1}^{2N}\left({\hat{x}}^{\left(i\right)}-\hat{x}\right){\left({\hat{x}}^{\left(i\right)}-\hat{x}\right)}^{\text{T}}$$

### 2.3. Correction Stage

When the output vector is a linear combination of the states, as described in Equation (8), there is no need to apply the unscented transformation again for the correction stage. The measurement update is computed with the asynchronous Kalman filter (AKF) Equations [29]:

(19) $${\hat{x}}_{\text{k}}^{+}={\hat{x}}_{k}^{-}+{\text{L}}_{k}\left({\text{y}}_{k}-\text{H}{\hat{x}}_{k}^{-}\right)$$

The symbol ${\hat{x}}_{k}^{-}$ corresponds to the *a priori* estimation at time *t_k_* (before the correction is performed) and ${\hat{x}}_{k}^{+}$ to the *a posteriori* estimation (after the correction).

The Kalman filter algorithm is geared to minimize the *a posteriori* estimation error covariance by finding the optimal value of **L***_k_* for each measurement update. It is calculated with the formula:

(20) $${\text{L}}_{\text{k}}={\text{P}}_{k}^{-}{\text{H}}^{\text{T}}{\left({\text{HP}}_{k}^{-}{\text{H}}^{\text{T}}+{\text{R}}_{k}\right)}^{-1}$$

The resulting *a posteriori* covariance matrix is:

(21) $${\text{P}}_{\text{k}}^{+}=\left({\text{I}}_{\text{N}}-{\text{L}}_{k}\text{H}\right){\text{P}}_{k}^{-}$$

where **I***_N_* is the *N*-th dimensional identity matrix. $P_{k}^{-}$ and $P_{k}^{+}$ are the a priori and a posteriori error covariance matrices, respectively (*i.e.*, the values of **P** before Equation (18) and after Equation (21), the measurement update). As can be deduced from Equations (20) and (21), the correction stage helps to reduce the trace of **P**, and the magnitude of the reduction depends on the precision of the measurement, which is given by the noise covariance matrix **R***_k_*.

## 3. Covariance-Triggered Measurements

The main requirement for an estimator algorithm is that it should have the capacity to provide an estimation with a small degree of uncertainty. When working with Kalman filters, this means that **P** must be bounded.

One idea for obtaining a bounded P would be to apply a measurement correction whenever the estimation error covariance matrix approaches an imposed threshold condition, which leads to an EBSE [23]. It has been observed that on linear systems with stationary noise, event generation converges to periodic sampling. In [24], this convergence is proven for scalar systems subject to specific conditions.

Moreover, if a periodic sampling is chosen, the problem becomes finding the appropriate sampling time that leads to the desired uncertainty level. To do so, a Riccati equation can be used to determine the steady-state value of **P**, and the detectability test of the system is a condition that ensures the existence of a positive-definite solution for that equation [28].

However, in the case of non-linear systems with variable noise parameters, a covariance-triggered EBSE does not converge to a periodic solution. The application of such an EBSE serves two different purposes. On the one hand, it maintains the estimation error covariance bounded. On the other hand, a sample is taken only when it is needed, so the use of sensors, network communications and processing resources is more efficient. In contrast to SoD methods, it is the estimator module (inside the vehicle), rather than the sensor that decides when to take a measurement (event generation).

The sampling intervals depend on the growth and initial value of the covariance matrix. The growth of **P** is independent from the sensed signals, so the next sampling instant can be calculated in advance by the estimator module. However, after applying the correction, the value of the estimation is influenced by the measurements, which, in turn, determines the dynamics of the prediction stage. This is why only the following time event, and not the subsequent ones, can be obtained in advance.

**Figure 2:**
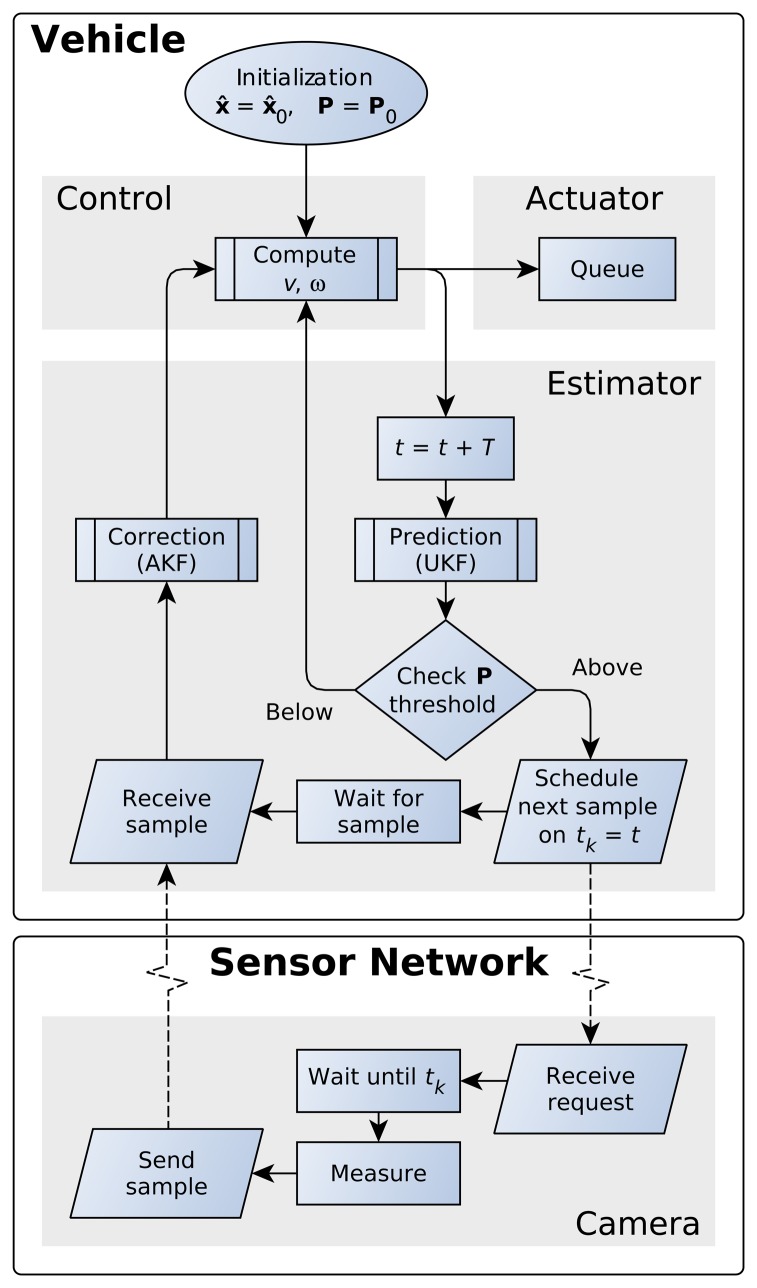
Flowchart of the event-based state estimator (EBSE), executed by the vehicle and the sensors.

The proposed EBSE algorithm is outlined in the flowchart shown in Figure 2.

With the estimated pose and the reference trajectory, the control module calculates the speed commands for the actuators (motors) with a guidance algorithm, such as [30,31] or similar. Using these commands, the estimator module can predict the location of the vehicle after *T* seconds and its error covariance. If the estimation error covariance remains below the threshold value, it is possible to calculate in advance the following speed commands, as well as the pose (after 2*T*, 3*T*, and so on). The speed commands are stored in a queue, so that they can be applied to the motion actuators at the right time.

This prediction process can be repeated until the estimation error covariance exceeds the threshold. When this happens, the estimated location is no longer sufficiently precise, and a measurement is required to reduce **P**. Thus, a measurement event is triggered for the time instant *t_k_*, where *t_k_* is the time instant when **P** will infringe on the triggering condition.

The estimator module then sends a request for a measurement at time *t_k_* to the corresponding sensor through the network. The sensor remains in a low energy state until *t_k_* and only switches on to take the measurement and send it to the vehicle, then switches off afterwards.

### 3.1. Distance Error and Orientation Error Thresholds

To design a covariance-triggered EBSE as described above, a condition for **P** must be chosen. In [25], each diagonal value was compared independently to a threshold value. A measurement was triggered every time any of the thresholds were exceeded. This condition ensures that the estimated error of each state remains at safe levels.

This paper presents a more intuitive and practical triggering condition. Instead of considering the errors of each state (coordinate) independently, it is more meaningful to have some knowledge about the location error as a distance to the real location. Dealing with the distance error is difficult, because it is a non-linear function of two correlated random variables. This is why it is easier to work with the squared error. Let $e_{d}^{2}$ be the squared distance error, defined as:

(22) $$e_{d}^{2}=e_{x}^{2}+e_{y}^{2}$$

The mean value of $e_{d}^{2}$ can be easily obtained from Equation (13).

(23) $$E\left[e_{d}^{2}\right]=E\left[e_{x}^{2}+e_{y}^{2}\right]=E\left[e_{x}^{2}\right]+E\left[e_{y}^{2}\right]=P_{1,1}+P_{2,2}$$

The square root of this value is known as the distance root mean squared error (DRMS), and it is a commonly-used indicator of localization precision [32].

(24) $$\text{DRMS}=\sqrt{E\left[e_{d}^{2}\right]}=\sqrt{P_{1,1}+P_{2,2}}$$

The probability of finding the real location within a ball centered on the estimated location and with a radius of DRMS for a Gaussian distribution is about 65%. The same ball, but with twice the radius (known as 2DRMS) raises the probability to 95%.

The estimation error of the orientation angle θ should also be taken into account. Accurate estimation of θ is critical for computation of the guidance control algorithm. Therefore, its own triggering condition is included to ensure that orientation uncertainty is always sufficiently small. The variance of the orientation angle error is given by the third element of the diagonal of **P**, as shown in Equation (12).

Although the orientation angle is not measured by the sensors, an observability test [33] on the non-linear system Equations (1) and (8) can determine that the system is locally observable if the linear speed *υ* is non-zero [34]. This means that every time the vehicle is moving, a measurement of position carries some information about the orientation and therefore can be used to reduce its estimation error variance.

By combining the distance and orientation error as mentioned above, the triggering condition for the sensors is the following: request a sample from a sensor iff:

(25) $$\left(P_{1,1}+P_{2,2}>D_{\text{thr}}^{2}\right)\vee \left(P_{3,3}>{\tilde{\theta }}_{\text{thr}}^{2}\right)$$

*D*_thr_ and *θ̃*_thr_ are threshold values that must be adjusted by the designer according to his or her needs. Thus, the expected DRMS would be lower than *D*_thr_, and more than 95% of the time, the distance error will be below 2*D*_thr_. Similarly, the orientation estimation error is expected to be around θ̃_thr_, with a 95% chance of being lower than 2θ̃_thr_.

The threshold values may not be constant and may vary along the route in order to adapt to the changing circumstances. In the following section, an adaptive threshold is proposed that takes into account the distance of the vehicle to the reference point.

### 3.2. Adaptive Distance Error Threshold

In the guidance problem, the vehicle must follow a reference track, but usually starts somewhere away from the initial position of this reference trajectory. The solution of the guiding problem can be divided into two different stages. First, the vehicle needs to approach the area of the reference trajectory and then follow it. The time during which the control algorithm is approaching the vehicle towards the trajectory is referred to as the approaching time.

When this task is completed, the vehicle is near the reference point and simply moves along the reference path. This stage is referred to as the tracking time.

During approaching time, the control algorithm does not need a very accurate estimated location. If the vehicle is far from the reference position, it is sufficient to have a rough idea of where it is, because the speed commands computed by the guidance algorithm would not differ greatly among the uncertainty region of the vehicle.

While approaching the trajectory, the radius of the uncertainty area (defined by the distance error threshold *D*_appr_) should be small compared with the distance of the vehicle to the reference point. This is easy to achieve if they maintain a fixed ratio of *K_D_*. In other words, during the approaching maneuver, *D*_thr_ can be set as:

(26) $$D_{\text{thr}}\approx \hat{L}K_{D}=D_{\text{appr}}$$

where *L̂* is the distance from the estimated position to the reference point.

**Figure 3:**
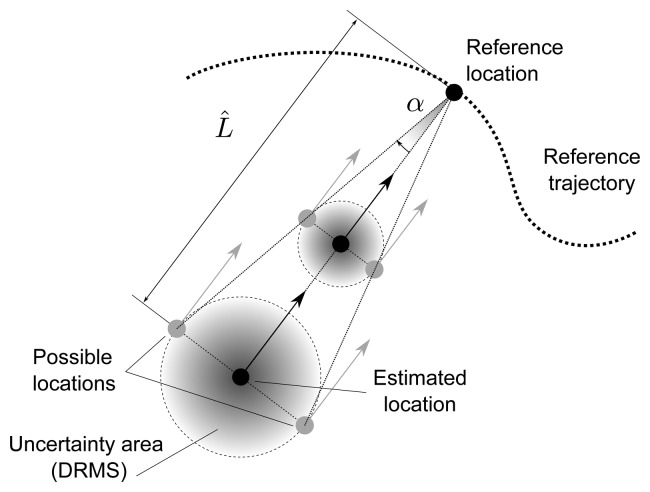
Diagram explaining the relation Equation (26) between the distance to the reference location and the size of the uncertainty region.

To understand the meaning of constant *K_D_*, let *α* be the angle between the estimated location and the limit of the uncertainty area as seen from the reference location (see Figure 3). Then,

(27) $$\alpha =\arctan\left(\frac{D_{\text{appr}}}{\hat{L}}\right)$$

Substituting Equation (26) into Equation (27) and solving for *K_D_* yields:

(28) $$K_{D}=\tan\alpha$$

The problem with a linear relation between *L̂* and *D*_appr_ is that the vehicle will move closer and closer to the reference trajectory, so *L̂* will tend to zero, and thus, *D*_thr_ will also tend to zero. An excessively low threshold leads to periodic sampling at the sensor's fastest sampling rate. The threshold should have a minimum value *D*_trk_. This parameter must be tuned according to the acceptable error during tracking time. The following smoothing function for the distance error threshold is proposed:

(29) $$D_{\text{thr}}=\sqrt{D_{\text{trk}}^{2}+D_{\text{appr}}^{2}}=\sqrt{D_{\text{trk}}^{2}+{\left(\hat{L}K_{D}\right)}^{2}}$$

**Figure 4:**
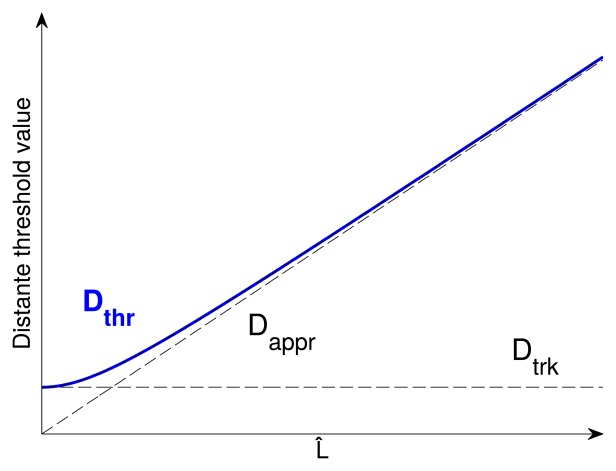
Adaptive distance threshold function *D*_thr_ as a function of the distance to the reference location.

This is a smoothing function that is close to *D*_appr_ when the reference point is distant (while approaching) and close to *D*_trk_ when the vehicle is near the reference point (while tracking). A graphical representation of the function is plotted in Figure 4.

Although the distance error is not critical during approaching time, orientation is still important. It is always critical to know where the vehicle is heading, even when it is far away from the reference point. Otherwise, any attempt by the control module to approach the desired point is not guaranteed to actually take it closer. This means that the threshold value for orientation error should remain a fixed value.

### 3.3. Limitations

A few assumptions are required to guarantee the correct operation of the proposed EBSE.

The first assumption is a perfect communication channel between the estimator and the sensors. However, it is possible to allow some delay in the transmission of the measurement packets, because the AKF can take care of them, even if they arrive out of sequence [29]. In other words, when the packet is received, the AKF can be applied to correct the estimation, even though it corresponds to a past time instant. Nonetheless, the covariance matrix would increase over the threshold until the packet with the measurement arrives.

The second assumption is that the only disturbances that affect the states are those modeled by input noise and its covariance matrix Σ_u_ (see Equation (3)). If, for example, the wheels slip on the ground, this will produce an estimation error, and the estimator will not react to it. As the estimator receives new measurements, the estimation will ultimately converge to the actual state, but the sampling times will not be adjusted to ensure a rapid correction of the estimation.

This is also a requirement for having an accurate **P** computed by the filter that truly reflects the estimation error covariance, which is the cornerstone of the proposed EBSE. As explained above, the UKF only computes an approximation of this covariance matrix, and the performance of the EBSE is tied to the accuracy that the filter can achieve on this approximation. Anyway, the UKF is well known for providing a good approximation of **P**, but nevertheless, in every practical scenario where the UKF does not work well, this EBSE will become impractical.

Finally, the third assumption is that the growth of **P** during the minimum acquisition time of the sensors is less than the reduction that such measurements can perform on **P**. If this assumption is not met, then even measuring at the fastest rate would not effectively reduce **P** over time. As a consequence, it cannot be guaranteed that P will remain below the desired limits. In this case, the estimator will request measurements from the sensors constantly, the sensors will provide samples at their fastest rate and the estimator will behave in a periodic fashion (where the time period is the minimum sampling time of the sensor). This problem arises when the threshold values are set too low, and therefore, the desired uncertainty cannot be met.

In order to determine the highest limits in DRMS and $\sigma_{\theta }^{2}$ (the variance of the orientation estimation error, *i.e.*, *P*_3,3_) that the system could be subjected to, it is possible to examine the worst-case scenarios where estimation uncertainty grows faster and the measurements have the highest possible noise covariance.

Different worst-case scenarios must be found for each of the two triggering conditions (DRMS and angle). Let:

(30) $$\begin{array}{cc} {\text{F}}_{\text{w}}=\frac{\partial f_{d}\left({\text{x}}_{\text{w}},{\text{u}}_{\text{w}}\right)}{\partial {\text{x}}_{\text{w}}}, & {\text{G}}_{\text{w}}=\frac{\partial f_{d}\left({\text{x}}_{\text{w}},{\text{u}}_{\text{w}}\right)}{\partial {\text{u}}_{\text{w}}} \end{array}$$

be the linearization matrices of the system for the worst-case scenario state point and inputs.

Assuming that the minimum acquisition time of the sensor is *T*_s_ = *MT*, where *M* ∈ ℕ, we define the equivalent matrices for a discrete system:

(31) $${\text{F}}_{\text{eq}}={\text{F}}_{\text{w}}^{M}$$

(32) $${\text{Q}}_{\text{eq}}=\sum_{\text{i}=0}^{M-1}{\text{F}}_{\text{w}}^{i}{\text{G}}_{\text{w}}\sum_{\text{u}}{\text{G}}_{\text{w}}^{\text{T}}{\left({\text{F}}_{\text{w}}^{i}\right)}^{\text{T}}$$

If the system stayed in these worst-case scenario conditions for a long time, it would be equivalent to a linear system, and therefore, matrix **P** would converge to a steady-state value that can be computed by solving a discrete-time algebraic Riccati equation (DARE):

(33) $${\text{P}}_{\text{w}}={\text{F}}_{\text{eq}}{\text{P}}_{\text{w}}{\text{F}}_{\text{eq}}^{\text{T}}+{\text{F}}_{\text{eq}}{\text{P}}_{\text{w}}{\text{H}}^{\text{T}}{\left({\text{HP}}_{\text{w}}{\text{H}}^{\text{T}}+{\text{R}}_{\text{w}}\right)}^{-1}{\text{HP}}_{\text{w}}{\text{F}}_{\text{eq}}^{\text{T}}{\text{Q}}_{\text{eq}}$$

where **R**_w_ is the worst measuring noise covariance matrix that the sensor can provide. The solution **P**_w_ of Equation (33) defines the maximum uncertainty that the EBSE could reach.

For each of the two triggering conditions (DRMS and angle), finding x_w_ and u_w_ for each case is an optimization problem: these are the points that lead to a **P**_w_, which has the maximum DRMS or $\sigma_{\theta }^{2}$, respectively.

Let *D*_w_ and θ̃_w_ be the values of DRMS and *σ*_θ_ of the worst-case scenarios mentioned above.

(34) $$D_{\text{w}}^{2}=\underset{\begin{array}{c} {\text{x}}_{\text{w}}\in \chi \\ {\text{u}}_{\text{w}}\in U \end{array}}{\max}{\text{P}}_{\text{w}}\left(1,1\right)+{\text{P}}_{\text{w}}\left(2,2\right)$$

(35) $${\tilde{\theta }}_{\text{w}}^{2}=\underset{\begin{array}{c} {\text{X}}_{\text{w}}\in \chi \\ {\text{u}}_{\text{w}}\in U \end{array}}{\max}{\text{P}}_{\text{w}}\left(3,3\right)$$

For the case under study, **u**_w_ for the DRMS condition is related to the maximum linear speed of the vehicle, and conversely, **u**_w_ for the $\sigma_{\theta }^{2}$ depends on the minimum linear speed. As stated above in this section, the orientation angle is an observable state as long as *υ* ≠ 0, so if the vehicle is not moving, θ cannot be estimated at all. Otherwise, the orientation and position uncertainties are correlated, and therefore, applying a position measurement update must reduce the orientation error variance. Additionally, the slower the speed, the less information can be drawn from a measurement, but provided that the vehicle moves with a guaranteed minimum speed, a solution to Equation (35) can be found.

If the triggering threshold values of Equation (25) are set to equal to or greater than *D*_w_ and θ̃_w_, then it can be guaranteed that the EBSE will maintain uncertainty below those bounds. This is true because the uncertainties will never grow faster than they do in the system represented by Equation (33), and the measurements will always have a better than or equal noise covariance matrix **R**_w_, but even under these worst-case conditions, the estimator is able to keep the uncertainties bounded.

These threshold values might be too high for some applications, and in general, the EBSE can perform better. However, in this case, the EBSE would work in a best-effort fashion where the uncertainty goal may not be achieved. Nevertheless, this uncertainty will be lower than or equal to *D*_w_ and θ̃_w_.

## 4. Illustrative Example

As a proof of concept, the proposed EBSE technique was tested in a simulation and compared to a periodic sampling estimator. To do so, the camera sensors were modeled and simulated, as well.

### 4.1. Simulation Setup

The tests were run with a discrete time step of *T* = 10 ms, as was the controller. The selected reference trajectory was a figure-eight shape described by:

(36) $$\{\begin{array}{ll} x_{r}\left(t\right)=5+4.5\sin\left(\frac{2\pi }{100}t+\frac{\pi }{2}\right) & \left[\text{m}\right]\\ y_{r}\left(t\right)=5+4.5\sin\left(\frac{4\pi }{100}t\right) & \left[\text{m}\right] \end{array}$$

The covariance matrix of the noise added to the inputs, explained in Equation (3), was:

(37) $$\sum_{\text{u}}=\left[\begin{array}{cc} 10^{-4} & 0\\ 0 & 10^{-2} \end{array}\right].$$

The vehicle implemented the Lyapunov-based guidance control (LGC) described in [30] for approaching and tracking the trajectory. This controller, based on the 2D non-holonomic unicycle system Equation (1), is intended for guiding a mobile robot when approaching and following a pre-programmed path.

The initial state vector of the vehicle was:

(38) $${\text{x}}_{0}=\left[\begin{array}{c} x_{0}\\ y_{0}\\ \theta_{0} \end{array}\right]=\left[\begin{array}{c} 7\\ 5\\ 0 \end{array}\right]$$

where the coordinate values are measured in meters and the orientation angle is in radians.

To ensure a short transient time for the estimator, the starting position of the robot is assumed to be known, with a small degree of uncertainty. This makes it possible to focus on the behavior of the estimator once it has converged to a value close to the real state. The initial estimation vector was identical to the real state vector, and the initial state estimation error covariance matrix was:

(39) $${\text{P}}_{0}=\left[\begin{array}{ccc} 0.1^{2} & 0 & 0\\ 0 & 0.1^{2} & 0\\ 0 & 0 & {\left(\frac{\pi }{6}\right)}^{2} \end{array}\right]$$

### 4.2. Camera Sensors

The sensors used to measure the location (*x* and *y*) of the vehicle were two cameras covering the working scenario. These cameras were simulated using the pin-hole geometric model [35] to imitate an inexpensive camera, such as the Unibrain Fire-i.

They were located at a height of three meters, pointing towards the ground at a 30° angle. The image resolution was 640 × 480 pixels, and the focal length was 4.3 mm. The minimum time between consecutive measurements taken by the cameras was 80 ms. The maximum rate of the sensor was therefore 12.5 frames per second (FPS). These cameras are able to deliver up to 30 FPS (33.3 ms of acquisition time), but the processing time of each frame must also be taken into account.

**Figure 5:**
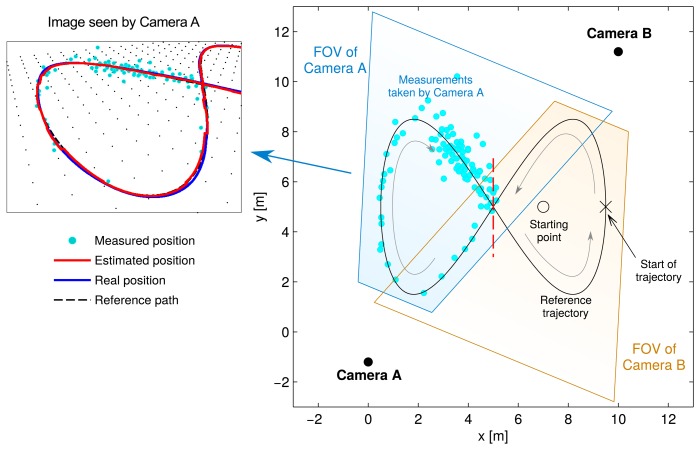
Diagram showing the reference trajectory and the camera locations. Each camera's field of view (FOV) is given.

Figure 5 shows the reference trajectory, the location of the cameras and each camera's field of view (FOV). Each camera covered one side of the figure-eight shape. Both cameras' FOV overlapped in the center, but for the sake of simplicity, only one of them was used at a time. The corresponding area for each camera is delimited by the red dashed line.

The non-linear transformation of the camera model could be performed by the UKF in the correction stage. However, in order to keep the estimator module independent from the kind of sensor technology used, it is assumed that the sensors are capable of delivering a position vector, such as Equation (8) and a noise covariance matrix **R***_k_*. Otherwise, the process of calibrating the cameras, or maybe substituting them with some other sensing technology, such as laser or ultrasound, would involve reconfiguration of the estimator.

The position of the vehicle is assumed to be determined by an image recognition algorithm that identifies the vehicle in a pair of coordinates (*U_k_*, *V_k_*) in the picture taken by the corresponding camera (e.g., [36]). To simulate the errors and deviations that the algorithm might make, zero-mean Gaussian random numbers Δ*U*_k_ and Δ*V*_k_ were added to each of the exact coordinates.

(40) $$\begin{array}{c} U_{k}^{'}=U_{k}+\Delta U_{k}\\ V_{k}^{'}=V_{k}+\Delta V_{k} \end{array}$$

In the above equation, *U_k_* and *V_k_* represent the exact point of the vehicle in the image and $U_{k}^{\prime }$ and $V_{k}^{\prime }$ represent the pixel coordinates that the simulated vision algorithm provides. Thus, the pixel in the image is related to a point on the floor (*z* = 0 plane) by the geometric equations of the camera model. The transformation of the point $\left(U_{k}^{\prime },V_{k}^{\prime }\right)$ results in vector **y***_k_*, which contains the noise **w***_k_*, as described in Equation (8).

The noises added to the two axes have a standard deviation of 12 pixels, and they are independent of each other. This yields the covariance matrix:

(41) $$E\left[{\left[\begin{array}{cc} \Delta U_{k} & \Delta V_{k} \end{array}\right]}^{T}\left[\begin{array}{cc} \Delta U_{k} & \Delta V_{k} \end{array}\right]\right]=\sum_{\text{i}}$$

(42) $$\sum_{\text{i}}=\left[\begin{array}{cc} 12^{2} & 0\\ 0 & 12^{2} \end{array}\right]$$

However, because the transformation of coordinates from the image to the world is a non-linear function, **w***_k_* has a covariance matrix **R***_k_* that is not diagonal and depends on the perspective of the point from the camera. This is small for points closer to the camera and becomes larger as the point moves farther away. **R***_k_* is calculated from **Σ**_i_ using the unscented transformation.

The bottom plot in Figure 5 shows how the measurements were simulated. It represents the scene as seen by one of the cameras. The black dots are intended to give an idea of the perspective of the ground. The distance between them is 50 cm.

Each cyan dot represents a measurement. They can be related one by one to the points in the top diagram to understand the effect of the perspective transformation performed by the camera.

This error magnitude is similar for every measurement, as seen in the picture. However, the measurements taken when the vehicle is close to the camera (in the lower half) are fairly accurate, whereas the ones corresponding to the upper half are not, in terms of distance in the real world. Consequently, a higher number of measurements are taken when the vehicle is moving at a distance from the camera, because the EBSE needs more of them to estimate its position with the same level of uncertainty.

### 4.3. Results

**Figure 6:**
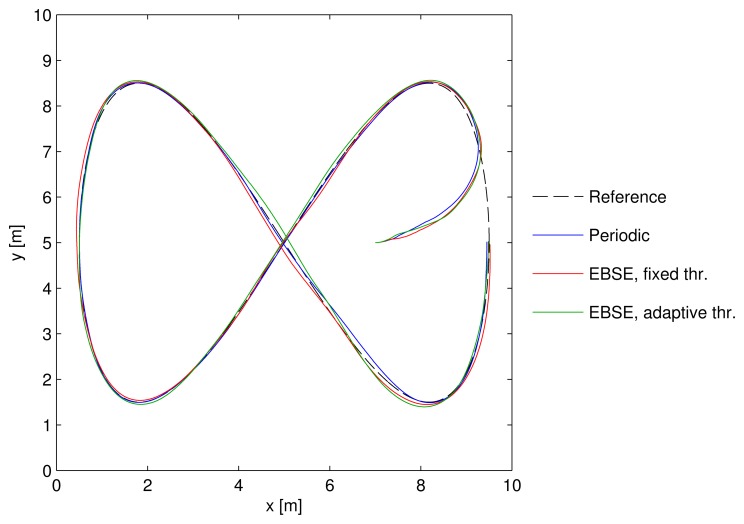
Comparison of the trajectory followed by the three sampling schemes, as well as the reference trajectory. The position of the camera sensors and their field-of-view (FOV) are also shown.

The results are presented as the comparison of three different cases. Figure 6 shows the trajectory followed by each strategy, and also the reference trajectory.

The three cases are:

(Blue) The UKF with periodic sampling. This represents the best possible estimation for the frame rate offered by the cameras.
(Red) The EBSE with a fixed distance threshold of *D*_thr_ = 75 mm.
(Green) The EBSE with the adaptive threshold described in Equation (29), where *D*_trk_ = 75 mm and *K_D_* = 1/6.

For the two last cases, the angle threshold was set to θ̃_thr_ = *π*/10 rad.

The periodic sampling strategy showed the best performance, because it used all of the information that the sensors could provide. Nevertheless, the other two methods also performed well, while only using a small fraction of the total number of measurements. The trajectories were within an error margin that would be acceptable for practical applications.

**Figure 7:**
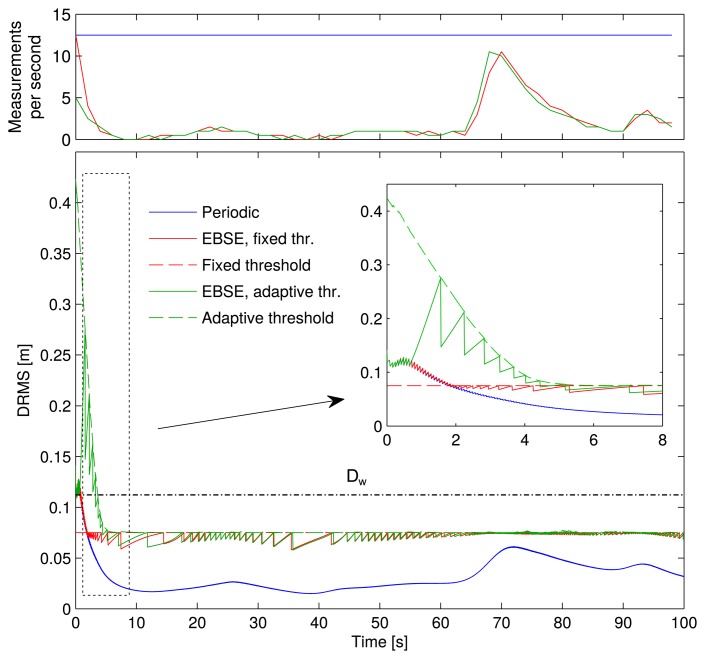
Measurement rate and evolution of the distance root mean squared error (DRMS) over time for the three estimation methods. The threshold values are also shown. The approaching time (until the eighth second of the simulation) is zoomed in the plot in the top left corner.

In order to compare the estimation error, the DRMS of each error is plotted in Figure 7, calculated with Equation (24). The top plot shows the number of measurements taken per second.

During tracking time (from *t* = 8 s onwards), the two EBSE showed the same behavior and maintained their DRMS below 75 mm.

The periodic sampling estimator had a smaller DRMS, but it increased up to 61mm in *t* = 72 s. When the vehicle was moving at a distance from the cameras (by the second half of the simulation time), their measurements contained more noise and, hence, provided less information to the estimator. As a result, this yielded greater uncertainty for the estimation.

This level of uncertainty was not far from the imposed threshold of the EBSE, and if it were considered tolerable, then it would be more efficient to reduce the use of the sensors whenever the estimation error was good enough.

The solution of Equation (34) for this case is *D*_w_ = 113 mm for a maximum speed *υ* < 0.7 m/s, and the solution of Equation (35) is θ̃_w_ = 0.082 rad for a minimum speed *υ* > 0.01 m/s. Since θ̃_thr_ > θ̃_w_, the expected angle uncertainty can be guaranteed. In contrast, *D*_trk_ is set below *D*_w_, and thus, the desired DRMS might not be achieved all of the time; but as the plot shows, in this case, it was achieved.

Within the DRMS plot, there is also a zoomed plot of the first eight seconds of the simulation. In the case of the fixed threshold EBSE, the estimator started with an initial uncertainty (**P**_0_) larger than the threshold, so it required as many measurements as it could obtain to reduce it quickly. The behavior was therefore identical to the periodic estimator until the DRMS dropped below the threshold. Then, a reduction in the use of the sensors began to take place.

However, the adaptive threshold EBSE only used a few measurements during approaching time. It obtained some at the beginning, triggered by the orientation threshold θ̃_thr_ in order to accurately determine the vehicle's orientation. Subsequently, very few measurements were required to locate the vehicle. The DRMS was very high compared with the other two methods, but it was still good enough to guide the vehicle towards the reference trajectory.

**Figure 8:**
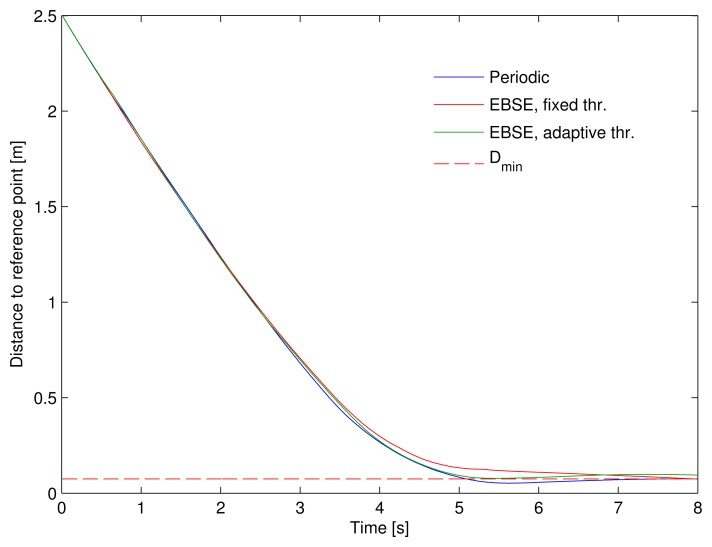
Distance to the point of the corresponding reference trajectory over time for the first eight seconds of the simulation (approaching time).

The approaching maneuver performed by the vehicle using the three different estimators can be compared in the trajectories shown in Figure 6 and also in the plot in Figure 8. This latter plot represents the distance of the vehicle to the corresponding point of the reference trajectory over time. The dashed line represents the minimum DRMS threshold of the EBSE estimators. Provided that the estimation error is somewhere around this value, the tracking performance is also limited by it.

The plot shows a very similar behavior for the three alternatives. In other words, the adaptive threshold EBSE's reduced use of the sensors did not imply a noticeable deterioration of the guidance during the approaching time.

Table 1 compares the performance of the three estimators during tracking time. The numbers shown are the average of 20 different simulations of each case. The estimation distance error column is the root mean square of the total distance error committed by the estimator for all of the tracking time. The position error column is the root mean squared distance between the real location of the vehicle and the corresponding point of the reference trajectory. The mean number of measurements taken is also shown.

As mentioned above, the effect of the adaptive threshold is hardly noticeable during tracking. However, Table 2 shows the results for the approaching time. The adaptive threshold EBSE halves the number of measurements compared with the fixed threshold EBSE, while the position error is very similar for all three cases.

## 5. Conclusions

This paper presents a combination of adaptive variance-based EBSE with a UKF that complements the guidance control of an autonomous vehicle whose position is detected by external sensors. Its use reduces the number of measurements taken without generating a deterioration in vehicle performance while performing approaching and tracking maneuvers. In addition, the desired DRMS of the estimation (which is one of the system parameters) is achieved. The results of the simulation tests confirm these conclusions and show that the number of measurements can be reduced to a small fraction of the total taken when using periodic sampling.

To implement the proposed algorithm, the remote sensor modules require limited intelligence: simply the capacity to respond to a vehicle request. In turn, they can be maintained in a standby state for most of the time.

The algorithm is tuned by adjusting three parameters: *D*_trk_, *K_D_* and θ̃_thr_, which are directly related to the desired estimation performance. Where the parameters are too restrictive and the desired performance cannot be met, the system would demand measurements from the sensors at their fastest rate. In this worst-case scenario, the EBSE would then simply become a periodic UKF.

## Figures and Tables

**Table 1:** Numerical results during tracking time (average results of 20 simulations).

	**Number of Measurements**	**Estimation Distance RMS Error (mm)**	**Position RMS Error (mm)**
Periodic sampling	1150	31.2	41.0
EBSE, fixed threshold	173.1	67.4	76.7
EBSE, adaptive threshold	170.8	68.9	78.5

**Table 2:** Numerical results during approaching time (average results of 20 simulations).

	**Number of Measurements**	**Estimation Distance RMS Error (mm)**	**Position RMS Error (mm)**
Periodic sampling	100	54.8	1028.5
EBSE, fixed threshold	35.6	76.4	1031.8
EBSE, adaptive threshold	17.6	102.4	1041.8
